# Cool Rooms

**Published:** 1870-09

**Authors:** 


### COOL ROOMS.

Open all the parlor windows and doors at daylight, and let them remain open at least until sunrise, then close them and darken the windows and they will remain delightfully cool and fresh for a great part of the day, besides keeping out the dust and flies; for want of this precaution many splendid parlors have a close sickening smell as you enter them, wholly incapacitating you from enjoying the beautiful things around you, and enjoying the visit to your friends. If the windows are opened after sunrise, the dust has begun to rise, and flies and other insects are all ready to soil the furniture, and everything else they light upon. Half the parlors of New York have not one good airing in a month.

